# The effect of goal frame and risk perception on digital flood prevention tool acceptability

**DOI:** 10.3389/fpsyg.2024.1454078

**Published:** 2024-09-18

**Authors:** Théo Jezierski, Isabelle Ragot-Court, Karine Weiss

**Affiliations:** ^1^Laboratory CHROME, Université de Nîmes, Nîmes, France; ^2^Laboratory LMA, Université Gustave-Eiffel, Salon-de-Provence, France

**Keywords:** flood, social acceptability, digital prevention, risk perception, goal framing theory

## Abstract

**Introduction:**

To cope with catastrophic floods, people need to be better prepared. In this context, a self-assessment digital tool for habitat vulnerability was developed. To improve its take-up rate, we are looking at the motivations associated with the social acceptability of this tool. The motivations (hedonic—gain—normative), derived from goal-frame theory, as well as elements relating to risk perception, are tested.

**Method:**

One, 688 participants (aged between 18 and 87) first read a scenario presenting the application (reflecting either one of the motivations of the Goal Framing Theory or a control scenario with no motivation). After reading one of the scenarios, they completed an online questionnaire, measuring the acceptability of the tool using three measures: a direct one (items from the Technological Acceptability Model: ease of use, perceived usefulness and social influence), an indirect measure (by asking the percentage of neighbours interested in the tool) and a social measure (judgement of a person using the tool). The last part of the questionnaire was about subjects’ risk perception.

**Results:**

The analyses show that, of all the scenarios, the one involving hedonic motivation leads to the lowest social acceptance of the tool. We also observe that a better risk perception predicts better tool acceptability. Finally, we observe interaction effects between risk perception and motivations, showing that normative motivation is better when risk perception increases and that the control condition is better when risk perception decreases.

**Discussion:**

Goal framing theory is usually used for ecological behaviors. It also appears here as relevant in the field of risk prevention. Although risk perception remains the best predictor of acceptability, these results lead us to conclude that hedonic motivation is not appropriate for the acceptability of a flood risk prevention tool. It is preferable to focus on normative and gain motivations.

## 1 Introduction

Climate change, combined with the development of prosperity, is leading to an overall increase in natural hazard losses. In France, for example, the cost of flooding is expected to increase by 81% in the period 2020–2050 compared with the period 1989–2019 (Lustman, 2021). These figures are in line with the latest report of the Intergovernmental Panel on Climate Change (IPCC), which states that flood damage will increase by up to 3.9 times if the temperature rises by 3°C (IPCC, 2022). However, people at risk do not always take the recommended preventive and protective measures, either at home or when travelling. In particular, knowledge of a risk does not systematically lead to awareness of that risk or to the adoption of preventive behaviour (Courant et al., 2021). Yet there are many information and prevention tools available, developed by both governments and private actors: official information campaigns, smartphone applications, risk prevention plans, etc. This raises the question of what determines the use and effectiveness of these tools. This study is accompanying the development of a digital application for self-diagnosing the vulnerability of individual homes to flooding^1^. In this context, we are seeking to understand what might encourage individuals to use this application. More specifically, we are interested in the motivations likely to be positively associated with risk prevention and, consequently, with the use of such a tool.

## 2 Flood risk

Although flooding is one of the most frequent natural hazards, its characteristics vary greatly from one area to another. For example, flash floods should be distinguished from slow-onset floods, even though both types are dangerous and devastating. Furthermore, some areas experience flooding relatively frequently, while others, although subject to this risk, have been spared for many years. These differences can make it difficult for people to understand the risk and assess their vulnerability to it. It is likely that these reasons (frequency and diversity) have led to extensive studies of flood risk perception in the literature (Birkholz et al., 2014; De Wolf et al., 2024; Faruk and Maharjan, 2023; Lechowska, 2018; Turner and Landry, 2023). The determinants of risk perception are increasingly well known in the social psychology literature. Recent studies have applied this knowledge to different fields and populations, such as Faruk and Maharjan’s (2023) study of farmers’ perception of risk. In addition, links have been made with other disciplines such as cognitive psychology, with De Wolf et al. (2024) studying risk perception through this less common prism. This shows that, in part, knowledge about risk perception is transferable in terms of scope and field of study.

Floods receive considerable media coverage (Verlynde, 2018), which suggest that they are part of everyday life (Scarwell and Laganier, 2004). Consequently, a process of normalisation May occur, whereby individuals tend to relativise their vulnerability in face of this risk (Luís et al., 2016). This also explains why flood risk is more easily forgotten than other risks (Verlynde, 2018, p. 121). It is therefore of great importance to assess the perception of flood risk in order to ascertain the most effective means of action, with the aim of enabling individuals to protect themselves as fully as possible.

The conceptual framework of the psychometric paradigm proposes three main factors inherent in the perception of risk: “Dread risk”, “Unknown risk” and “Exposure” (Slovic et al., 1980). The first factor corresponds to the degree of fear aroused by the risk: the greater the fear, the higher the risk perception. The second factor is the extent of people’s knowledge regarding the risk in question. It is positively correlated with risk perception. Lastly, exposure corresponds to the number of individuals exposed to the risk: the greater the number, the greater the perception of the risk. A multitude of determinants influences these factors. Although some factors are applicable to all types of risk, others are specific to particular risk categories. The experience of risk is an important factor in the perception of flood risk. A multitude of studies have demonstrated that the experience of a flood increases the perception of risk (Botzen et al., 2009; Burningham et al., 2008; De Wolf et al., 2024; Faruk and Maharjan, 2023; Miceli et al., 2008; Zhang et al., 2010). Ruin et al. (2007) also indicate that individuals who have not experienced flooding tend to underestimate this risk, whereas those who have do overestimate it. Nevertheless, some authors argue that direct experience can mitigate the perception of risk when the risk has not yet produced tangible negative consequences (such as damage to individuals or buildings) or when it occurs with infrequency (Burningham et al., 2008; Scolobig et al., 2012). Conversely, direct experience with negative consequences has been found to positively affect risk perception (Siegrist and Gutscher, 2006). It is also important to highlight the positive correlation between personal experience and the implementation of protective behaviours. Individuals who have previously experienced flooding are more likely to adopt protective behaviours than those who have not (Lemée, 2017). However, the relationship between risk perception and protective behaviour is less straightforward. In some studies, there is a positive correlation between risk perception and the probability of taking protective behaviours, while in others, the opposite is observed (van Valkengoed and Steg, 2019; Wachinger et al., 2013). Similarly, there is no evidence that a high-risk perception necessarily leads to the adoption of protective behaviours (Paton et al., 2000). However, risk perception does influence motivation to protect oneself (Grothmann and Reusswig, 2006).

One factor that can influence the preparation behaviours in other contexts (financial crisis and vaccine) is their social acceptability (Fossati and Trein, 2023; Nakamanya et al., 2022). Indeed, if individuals perceive that performing these behaviours is not socially valued, then not performing them avoids social sanction. Consequently, it is imperative to investigate the *a priori* social acceptability of prevention measures in advance in order to ascertain whether they will be employed by individuals.

In this study, prevention behaviour depends on the acceptability of a digital flood prevention tool. In our particular case, we cannot take “use” of the application as the prevention behaviour, as it is still being developed. We have therefore chosen to focus on the tool’s *a priori* acceptability. This allows us to measure what people think of the tool before it is finished. We therefore want to see whether the acceptability of this tool by the population is influenced by the perception of risk.

## 3 *A priori* social acceptability of the technology

The *a priori* social acceptability of this digital application is of interest, defined as “the subjective representation of the use of the technology” (from French, Terrade et al., 2009). In the context of *a priori* social acceptability, individuals are required to judge the object without being able to manipulate it. This is measured through the evaluation of the object by potential users (Lefeuvre et al., 2008). In this manner, individuals are prompted to assess the devices prior to their design and utilisation, thereby enabling the identification of those that will be most readily accepted and, consequently, most likely to be utilised. It is insufficient to be convinced of the usefulness and effectiveness of a system in order to use it (Lefeuvre et al., 2008). This brings us back to the more classic work in social psychology, which demonstrates that the relationship between attitude and behaviour is not linear (Ajzen, 1991). Furthermore, social and normative constraints, as well as the need for control, can influence the utilisation of the object (Lefeuvre et al., 2008). It is therefore necessary to consider the social aspect, which has the potential to impact the acceptability of the object.

According to the Technology Acceptability Model (TAM), which appears to be the most commonly used in the literature (Shin, 2009; Stiegemeier et al., 2022; Sykes et al., 2009), perceived usefulness and perceived ease of use are two factors that help to explain the intention to use a technology (Davis, 1989). Perceived usefulness can be defined as “the intensity of an individual’s belief in the potential benefits of using technology to enhance performance in a professional or organisational context” (from French, Pasquier, 2012, p. 40). The concept of perceived ease of use can be refers to the “user’s perception of the simplicity of the technology in question. This perception is based on the user’s belief that using the technology requires little or no effort” (from French, Pasquier, 2012, p. 40).

Nevertheless, Lefeuvre et al. (2008) emphasise the social dimension of *a priori* social acceptability, particularly the potential pitfall of a normative judgement that could be associated with the representation of a technology. In essence, the technology must be evaluated not on the basis of the benefits it is likely to confer, but on the manner in which it is employed. For instance, presenting a tool designed to reduce vulnerability to risk is likely to generate considerable support, due to its social desirability. This does not prejudge the acceptability of its use by the individuals concerned. It is challenging to envisage a pro-social tool whose objective is to protect individuals being considered as useless. We therefore drew inspiration from the work of Lefeuvre et al. (2008), who proposed a measure of social acceptability designed to avoid such normative responses. The measure in question enquires as to how a person utilising the application would be perceived by their family, friends and neighbours. By focusing on the individuals using the technology, rather than the technology itself, we can gain a more nuanced understanding of the social judgments that shape its acceptability.

## 4 Goal framing theory

The motivation to use a tool is also a significant factor in determining its acceptability (Huijts et al., 2012). Consequently, it is possible to associate tool use with additional motivations that serve to reinforce the intention to act. This is based on the goal framing theory, which posits that three overarching goals form the basis of motivation to act: normative, hedonic, and gain goals (Lindenberg and Steg, 2013). While the final two goals are egocentric, the normative goal is collectively oriented. It refers to acting in an appropriate manner and involves the activation of a collective identity. In this sense, it is the most sensitive to social context. The gain goal is concerned with the preservation and nurturing of personal resources, which May be financial, material or related to social status. The hedonic goal is oriented towards the immediate satisfaction of basic needs and the pursuit of immediate well-being. It is naturally the most salient, and therefore the least context-sensitive (Lindenberg, 2001). The theory posits that these three fundamental goals are of paramount importance and that their dynamics are of significant interest. It is proposed that when one goal is salient, the other goals are always present in the background. When an individual pursues a goal, it becomes a primary concern, activated, and exerts influence over the individual’s thoughts, information selection, and the possibilities of action available to them. Consequently, he becomes more attuned to the cues associated with this goal. To illustrate this point, consider the example provided by Lindenberg and Steg (2013). When we are hungry, our attention is drawn to the sensory and cognitive cues associated with edible elements, while other information, such as the price or the long-term effects of the food we desire, is likely to be ignored. Such information becomes irrelevant, distracting, or even contradictory to the main goal that has been activated. Nevertheless, it is possible for multiple goals to coexist in a single individual, as evidenced by the literature (Frederick et al., 2002). However, the majority of studies focus on examining a single goal at a time (Bamberg and Schmidt, 2003; Stern, 2000). Furthermore, certain factors are likely to influence the salience of one of these goals. In this sense, the normative goal is the most sensitive to the social context, that is to say to what others are doing.

Goal framing theory has been developed in the field of social and environmental psychology, with the primary objective of providing a framework for understanding the adoption of pro-environmental behaviours (Lindenberg and Steg, 2013; Yang et al., 2021). However, it has also been applied in other fields, such as organisational governance (Birkinshaw et al., 2014). The authors have primarily concentrated on the means of reinforcing the normative goal in order to maintain its salience in the face of hedonic and gain goals. It is argued that the normative goal, through its collective dimension, is most likely to positively influence pro-environmental behaviours.

To the best of our knowledge, this theory has never been applied in the field of natural disasters, and consequently has never been used to understand the motivations for adopting preventive behaviours in the face of flood risk. Nevertheless, this transposition of the ecological issue to that of flood risk prevention appears to be a pertinent one, given that both involve behaviours that help to reduce vulnerability to an environmental risk.

## 5 Objectives

In order to understand how to encourage the use of a digital application, we want to know whether the salience of the framework’s objectives can be activated and have an impact on the evaluation of the application.

The research is informed by five main hypotheses:

- Firstly, we assume an effect of socio-demographic variables. More specifically, we assume that only direct experience of the negative consequences of a flood will have a psychological effect on the acceptability of the application. Conversely, having experienced a flood with no negative consequences will reduce the acceptability of the tool (H1).
- Second, we hypothesise that the perception of risk will predict acceptability of the digital application: the greater the perceived risk, the greater the acceptance of the tool (H2).
- Third, we hypothesise that the activation of the salience of the goals frame will result in a better acceptability of the application, comparing to the absence of the activation of the salience of the goals frame (H3).
- We also expect an interaction effect between the goals and the perception of risk on the acceptability of the tool. We hypothesise that participants will better accept the use of the application more when one (or more) goal(s) frame are activated and when their perception of risk is high, rather than when their perception of risk is low (H4).
- In general, we assume that the normative goal will result in more acceptability because it is more sensitive to context, as Lindenberg (2001) posits (H5).

## 6 Materials and methods

### 6.1 Scenario pre-tests

For this study we want to use a scenario-based method. In other words, we want to ask participants to read a short text presenting the digital prevention tool and then evaluate it. However, we need to pre-test these descriptions, each of which contains one or more motivations presented by the framework of goal theory (hedonic—gain—normative—gain and normative) and a control description. An initial pre-test was conducted with a sample of 106 individuals, comprising 46 women, 16 men, and 3 individuals who declined to respond. The mean age of the sample was 31.1 years (SD = 11.7), with a minimum age of 19 years and a maximum age of 80 years. In order to maintain a sufficient number of respondents, we elected to include individuals who had not completed the socio-demographic information but who had nevertheless answered the questions. Each participant was first randomly confronted with a scenario. After reading it, they were asked to rate the clarity and comprehensibility of the text on a 5-point Likert scale. Subsequently, the participants were asked to rate the motivations associated with using the application on a 5-point scale. These included whether it was funny (hedonic goal frame), whether it would result in financial gain (gain goal), and whether it would be used by a large number of people (normative goal). We then asked participants what would be the best things to say to make the application make more money (gain), to make it seem more fun (hedonic), to make it clear that a lot of people are interested in it (norm). Subsequently, data pertaining to the participants’ sociodemographic characteristics, including age, gender, postcode, town, and socio-professional category, was collected. We then carried out ANOVAs to determine whether the scenarios did indeed represent the expected motivations and whether, compared with the other scenarios, the desired motivations were indeed the highest.

Based on the recommendations of the first participants, we modified the scenarios and tested them with a second pre-test of 106 participants. This sample was made up of 63 women, 31 men, 3 who selected the “other” modality and 4 no response. The average age was 28.3 (SD = 9.3), with a minimum of 18 and a maximum of 59. The pre-test procedure was the same as for the first test. We then carried out tests identical to the first pre-test (ANOVAs) to check that the scenarios induced the desired motivations.

The hedonic goal proved to be the most challenging to operationalise in conjunction with the digital application pertaining to flood risk. Moreover, following the first test of the scenarios, it became evident that the hedonic motivation was the least well-received by the participants.

The results of the preliminary tests are presented in Table 1.

**Table 1:** Results for scenarios retained after pre-testing, by scenario.

	Funny	Financial gain	Used by a lot of people
Hedonic	3.50	2.33	2.39
Gain	3.00	3.82	2.65
Normative	2.46	2.06	3.65
Gain + normative	2.93	3.00	3.60
Control	3.00	3.07	3.07

### 6.2 Material and procedure

Following the completion of the material tests, a questionnaire is produced, comprising four sections, plus an information leaflet about the study, together with a consent form guaranteeing anonymity and confidentiality of information. The research complies with the country’s ethical rules. The questionnaire was distributed online in November 2022.^2^ All the participants who completed the questionnaire in full were included in the study. It should be noted that participants were permitted as much time as they wanted to complete the questionnaire. Furthermore, no remuneration was provided to participants. In order to ensure the greatest possible representativeness, we requested that the panellist be distributed in a manner that reflected the gender and age demographics of the larger French population. There were no exclusion criteria except age (subjects had to be more than 18th years old) and the comprehension of French language.

Firstly, participants are asked to provide information regarding their socio-demographic characteristics. The questionnaire includes questions regarding the participants’ age, gender, and place of residence. These includes town of residence, type of residence (house or apartment), length of residence, and tenure status (owner, tenant, living rent-free, or other). The subsequent section of the questionnaire pertains to flood risk and experiences related to floods. This includes questions regarding the occurrence of floods in the neighbourhood, the date of the most recent flood, direct experience of floods and the most recent date, direct experience of negative consequences of this/these floods (on property, housing, pets, the respondents themselves and those around them), and experience of flooding by one or more relatives (Lived or not lived).

The second part of the study concerns the presentation of the digital application. It begins with the presentation of one of the five scenarios from the pre-test phase. All of the scenarios share a common structure, presenting a digital tool such as a flood risk prevention application. The scenarios are differentiated according to the goal frame that is activated.

- Hedonic scenario (190 words) presents the digital application as a game: “Flooding is a serious issue, but why not try learning the right ways to deal with it while having fun! That’s what the MAIF Foundation and a team of researchers want to offer you by developing a free mobile/tablet game.” (Extract of the hedonic scenario. Translated from French).
- Gain scenario (122 words) suggests that participants use the application to acquire knowledge and discounts: “This will also give you access to advice and links to help you benefit from equipment and even fittings at a very low cost! Up to 80% reimbursement! This means you can acquire new equipment at a lower cost, so you can protect yourself and your property.” (Extract of the gain scenario. Translated from French).
- Normative scenario (120 words) indicates that a lot of people use the digital application: “Easy to access, this application can be used by as many people as possible to help protect everyone. In fact, surveys show that 80% of those questioned are ready to use it.” (Extract of the normative scenario. Translated from French).
- Gain + normative scenario (192 words) combines the previous two: “Stay informed, gain know-how and enjoy financial benefits thanks to this application. What’s more, together we can help those involved in flood management, particularly firefighters and rescue workers.” (Extract of the gain + normative scenario. Translated from French).
- Control scenario (190 words) just gives information about the digital application: “This application describes the behaviour you should adopt before and during a flood, and explains why it’s important. Depending on how much time you have before the water arrives: there’s always something to do! Whether you are at work, in the car or at home, you’ll find useful and appropriate advice!” (Extract of the control scenario. Translated from French).

After reading one scenario, participants are asked to assess the acceptability of the application. Acceptability is measured with 3 kinds of indicators: social acceptability, direct acceptability and indirect acceptability. Frist, in accordance with the methodology proposed by Lefeuvre et al. (2008), we evaluated the social acceptability of individuals utilising the digital. The question is as follows: “In your opinion, the extent to which an individual who knowingly chooses to use this tool would be perceived as responsible in the face of the risk of flooding by his family, friends, or neighbours” (*From French*) is to be rated on a scale ranging from −3 “Not at all responsible” to +3 “Completely responsible” (*α* = 0.90). This measure limits the bias that would result from direct measurement by asking individuals to evaluate an individual using the tool rather than the tool itself. Second, in order to assess the direct acceptability, we drew upon the Technology Acceptance Model (TAM), as proposed by Kamal et al. (2020). Items were drawn from three factors deemed relevant to the study’s object of interest: ease of use (e.g., *I believe it would be simple for me to comprehend how to utilize this application*), perceived usefulness (e.g., *This application enabled me to cope more effectively with the risk of flooding*), and social influence, defined as “the degree or the extent to which a person believes that others, especially, his/her acquaintances and friends believe that he/she should use a new” (Kamal et al., 2020) (e.g., *It seems reasonable to posit that the individuals with whom I interact on a regular basis (friends, family, and acquaintances) would be in favour of my using this application*.). The first two factors are found in numerous theoretical conceptions of acceptability (Chao, 2019; Stiegemeier et al., 2022; Venkatesh et al., 2003). Third, the judgement is measured indirectly by requesting that respondents indicate the percentage of their neighbours who would use this application (from 0 to 100%).

The final section of the questionnaire assesses the perception of risk by adapting the scale developed by Lemée (2017), which originally focused on the perception of marine submersion risk. This measure comprises four factors, which can be grouped according to the psychometric paradigm (Slovic, 1990). These are: fear and stress aroused by the risk (which we group together under the term “affects”), knowledge of the risk, exposure to the risk and collective vulnerability. Our adaptation of the scale includes 13 items translated and adjusted to the flooding context (the initial scale focused only on the coastal flooding issue). We also pre-tested the clarity and comprehensibility of the French items with the same first sample of scenarios. We identified with two factorial analysis (exploratory and confirmatory) a three-factor structure: exposure (*α* = 0.817), affects (*α* = 0.778) and knowledge (*α* = 0.532). Despite a low alpha for the knowledge dimension, we have chosen to retain this factor in order to propose a measure that is consistent with the elements emerging from the literature. The participants were requested to indicate their level of agreement on a scale ranging from 1 (strongly disagree) to 5 (strongly agree). By placing this variable at the end of the questionnaire, we can control for its potential impact on the perception of the scenarios and the salience of the activated goal frame. The effect of scenario perception on this variable will be statistically controlled.

### 6.3 Participants

The online questionnaire was distributed in four regions of mainland France (Occitanie, Provence-Alpes-Côte d’Azur, Auvergne-Rhône-Alpes and Île-de-France, with the exception of Paris city center). The regions were selected on the basis of their inclusion of areas at significant risk of flooding^3^ (^4^). These areas correspond to significant human, economic and social stakes subject to a significant risk of flooding. Furthermore, all of these regions have been subject to numerous natural disaster decrees pertaining to flooding. The Mediterranean regions (Occitanie and Provence-Alpes-Côte d’Azur) are also regularly subject to intense Mediterranean phenomena (Lebriton, 2022).

A total of 1,688 participants completed the questionnaire. The sample comprised 53.4% female participants. The participants were aged between 18 and 87 years (M = 47.2, SD = 16.1). The distribution of participants across the five scenarios is equitable, with an average of 337 participants per condition. Our sample consists of 312 participants who have experienced at least one flood (1,376 have not). In addition, 615 participants rent their accommodation, 966 own their own home and 107 are housed free of charge. Ultimately, 767 individuals reside in a flat, while 921 individuals live in a house.

## 7 Results

### 7.1 Manip check

We test the scenarios with an analysis of variance (ANOVA) to verify their possible effect on risk perception. We observe no difference between the scenarios on the Exposure factor *F*(4, 841) = 1.702, *p* = 0.147; on the Affects factor *F*(4, 841) = 0.966, *p* = 0.425; and on the Knowledge factor *F*(4, 841) = 0.726, *p* = 0.575. Our scenarios therefore have no effect on risk perception.

### 7.2 Sociodemographic influences

Our first hypothesis concerns the effect of flooding experience on acceptability: experiencing a flood with negative consequences would improve the tool’s acceptability, whereas experiencing one without would reduce it. To test it we conducted a Fisher and Welch ANOVA to compare four groups:

- The group without experience of flood (*N* = 1,376).
- The group with indirect experience of flood, i.e., people who had experienced flooding in their neighbourhood without being affected (*N* = 91).
- The group with direct experience without consequences (*N* = 73).
- The group with direct experience with negative consequences (*N* = 148).

**Figure 1:**
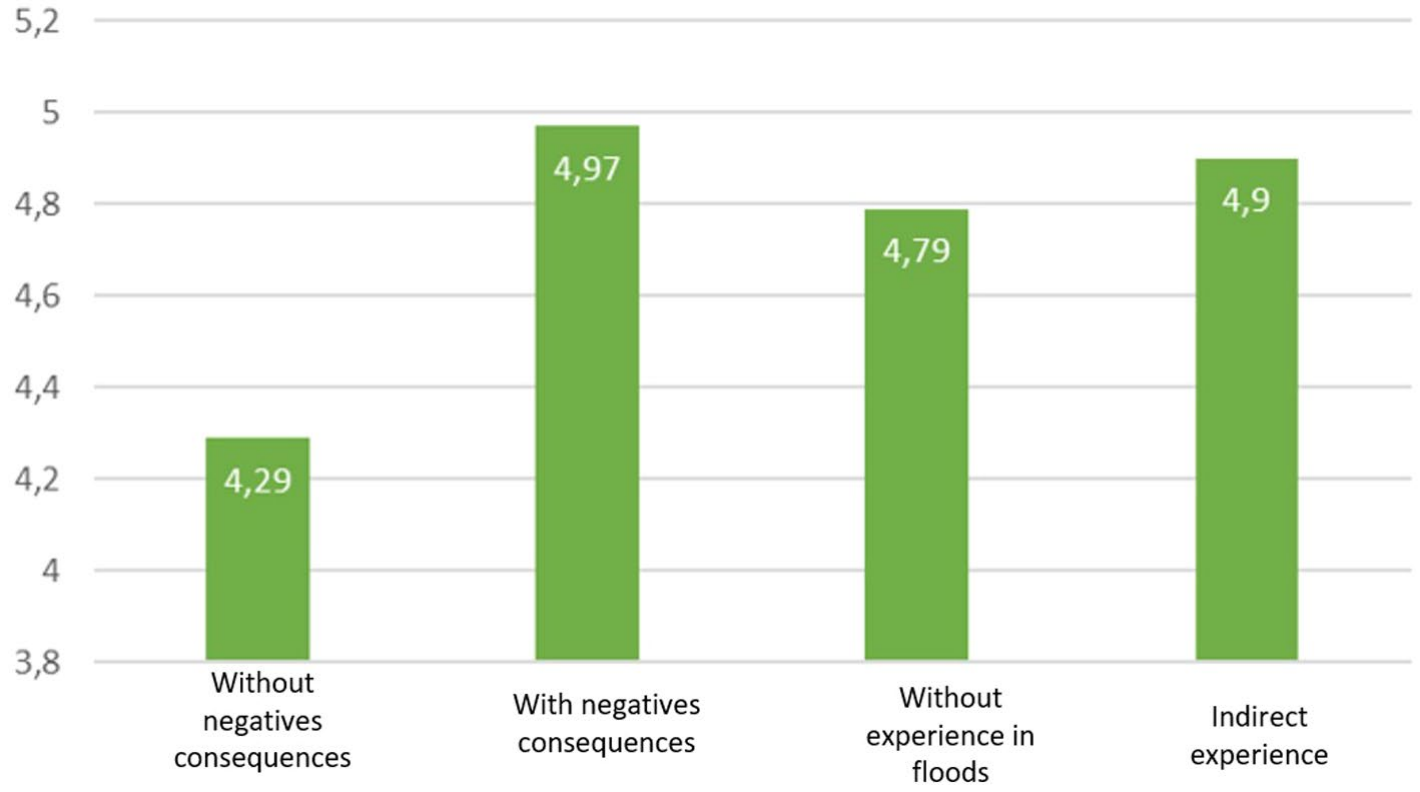
Mean of direct acceptability as a function of flood experience.

A significant difference is observed between the groups for the direct judgment (*F* (3, 1,684) = 3.52, *p* = 0.015, *η*^2^ = 0.006), and for indirect judgment (*F*_Welch_(3, 182) = 3.51, *p* = 0.016) but not for the social judgment (*F* (3, 1,684) = 1.38, ns). Tukey and Games-Howell pairwise comparisons are conducted to ascertain the specific locations of these differences. Significant differences are observed between the group with direct experience without consequences (M = 4.3, SD = 1.5) Vs. the group with direct experience with negative consequences (M = 5.0, SD = 1.6), *t*_Tukey_(1684) = −3.15, *p* = 0.009 and the group without experience of floods (M = 4.8, SD = 1.5), *t*_Tukey_(1684) = −2.73, *p* = 0.033. There is a marginal difference with the group with indirect experience of flood (M = 4.9, SD = 1.4), *t*_Tukey_(1684) = −2.555, *p* = 0.052. For the direct judgment, having experienced flooding without negative consequences would reduce the acceptability of the tool. For the indirect judgement, there is a significant difference between the group with indirect experience of flood (M = 39.6, SD = 23.9) and the group with direct experience without consequences (M = 28.2, SD = 22.1), *t*_Game-Howell_(159) = −3.15, *p* = 0.010 (cf. Figure 1).

With regard to relatives, we perform a Student’s and Welch’s test, which reveal differences between participants whose relatives had experienced flooding (*N* = 650) and those whose relatives have not (*N* = 1,038) on the three judgment scores: direct *t*_Welch_ (1488) = −5.39, *p* < 0.001, *d* = −0.266; indirect *t*(1686) = −6.04, *p* < 0.001, *d* = −0.302; social *t*_Welch_(1431) = −3.04, *p* = 0.002, *d* = −0.151 (cf. Table 2).

**Table 2:** Mean of judgement scores according to relatives’ experiences.

Judgement\Relatives’ experience	Lived	Not lived
Direct acceptability (score de 1 à 7)	5,01	4,62
Indirect acceptability (score de 0 à 100)	38,97	31,3
Social acceptability (score de-3 à +3)	1,76	1,58

Our first hypothesis is partly validated. People who have experienced flooding without any negative consequences are less likely to accept the application. However, those who have experienced negative consequences do not increase their acceptability compared with those who have not.

### 7.3 Risk perception and acceptability

We also assume that risk perception will be a predictor of the tool’s acceptability (H2). We run a multiple linear regression to test the predictive effect of risk perception factors on the three acceptability measures. The model is significant for direct acceptability [*F*(3, 1,684) = 149, *p* < 0.001, *R*^2^ = 0.210]. The exposure and affect factors are predictive of acceptability [*t*(1684) = 8.00, *p* < 0.001, *β* = 0.190; *t*(1684) = 15.36, *p* < 0.001, *β* = 0.352] while the knowledge factor is not [*t*(1684) = 1.34, *p* = 0.181, *β* = 0.003].

These results are similar for social acceptability. The model is significant *F*(3, 1,684) = 78.93, *p* < 0.001, *R*^2^ = 0.122. The exposure and affect factors are predictive of individuals’ social acceptability [*t*(1,684) = 11.22, *p* < 0.001, *β* = 0.281; *t*(1,684) = 6.22, *p* < 0.001, *β* = 0.150] whereas the knowledge factor is not predictive [*t*(1,684) = −1.044, *p* = 0.299, *β* = −0.025].

Finally, for indirect acceptability the model is significant *F*(3, 1,684) = 62.9, *p* < 0.001, *R*^2^ = 0.10. In this case, the affect and knowledge factors are predictive [*t*(1,684) = 12.03, *p* < 0.001, *β* = 0.294; *t*(1,684) = 3.67, *p* < 0.001, *β* = 0.089] whereas the exposure factor is not [*t*(1684) = −0.029, *p* = 0.772, *β* = −0.007].

Our second hypothesis is confirmed. Risk perception, in particular exposure and affect factors, predict the acceptability of the digital prevention tool.

### 7.4 Relevance of frame goals and acceptability

The hypothesis was that scenarios with salient goals frame would be linked to more acceptability comparing to the control scenario (H3). To test this hypothesis, an ANOVA is carried out on each of the acceptability scores (direct judgement, indirect judgement, social judgement).

**Figure 2:**
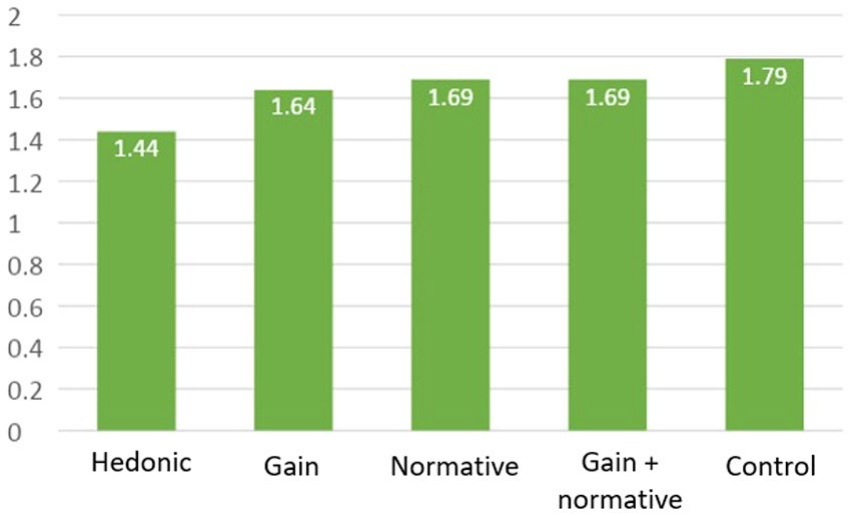
Mean of social judgement according to scenario.

The results indicate that there is no significant difference in direct judgement according to the five scenarios proposed (*F*(4, 1,683) = 0.43, *p* = 0.785) or in indirect judgement (*F*(4, 1,683) = 1.65, *p* = 0.160). Conversely, significant differences are observed between the different scenarios in social judgement [*F*(4, 1,683) = 4.25; *p* = 0.002; *η*^2^ = 0.010]. See Figure 2.

Tukey’s pairwise comparison test reveals that the Hedonic scenario (M = 1.44, SD = 1.20) results in a significantly lower social judgment of the tool than the scenarios:

- Normative (M = 1.7, SD = 1.2), *t*_Tukey_(1683) = −2.76, *p* = 0.046.
- Gain + Normative (M = 1.7, SD = 1.2) *t*_Tukey_(1683) = 2.85, *p* = 0.036.
- Control (M = 1.8, SD = 1.1), *t*_Tukey_(1683) = −3.95, *p* < 0.001.

The Gain scenario (M = 1.6, SD = 1.2) do not differ significantly from the Hedonic scenario: *t*_Tukey_(1683) = 2.28, *p* = 0.152.

Our third hypothesis is in partly confirmed. Motivation seems to have an impact on acceptability. However, they are not necessarily more useful than the control scenario. On the other hand, hedonic motivation is not the most appropriate way of improving acceptability.

### 7.5 Interaction between salience of goals frame and perception of risk on acceptability scores

We test an interaction effect between the scenarios and the perception of risk on the acceptability measures.

**Figure 3:**
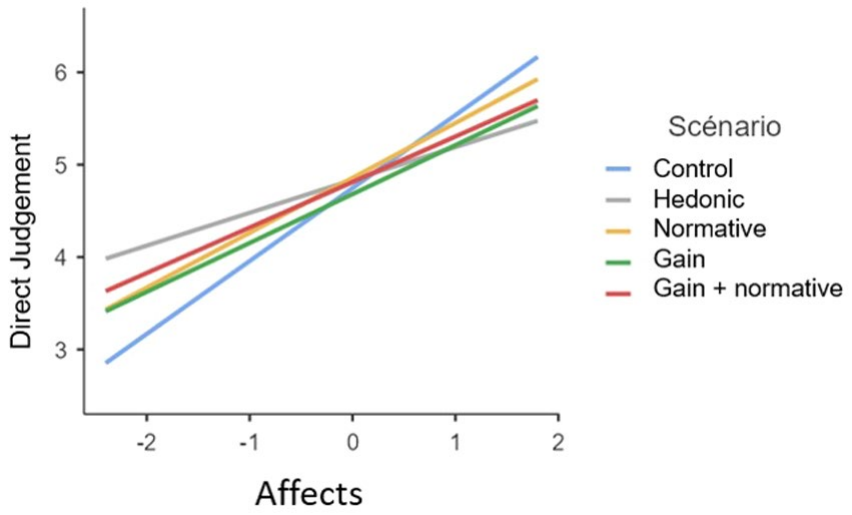
Graph of the interaction between the affect factor and the scenarios on direct acceptability.

Firstly, for indirect acceptability, we observe no interaction effect. Secondly, for direct acceptability, we observe an interaction effect between affects and scenarios (see Figure 3). Here, we chose to use the “Control” modality as the reference modality. We obtain the following results:

- Gain and Control, [*t*(1668) = −2.27, *p* = 0.023, *β* = −0.169].
- Gain + Normative and Control [*t*(1668) = −2.59, *p* = 0.01, *β* = −0.194].
- Hedonic and Control [*t*(1668) = −3.80, *p* < 0.001, *β* = −0.283].
- Normative and control [*t*(1668) = −1.72, *p* = 0.086, *β* = −0.127].

Consequently, in each of the scenarios with motivation, the regression coefficient is observed to be lower than in the control scenario. Figure 3 illustrates that participants with low affect towards flooding who have viewed the control scenario tend to rate the tool more negatively than those who have viewed the other scenarios with motivation. In other words, when affect is low, the use of motivations makes the tool more acceptable than the control scenario in terms of direct judgement. Conversely, when affect levels rise, the control scenario has a greater impact on direct judgement of the tool than the other scenarios.

**Figure 4:**
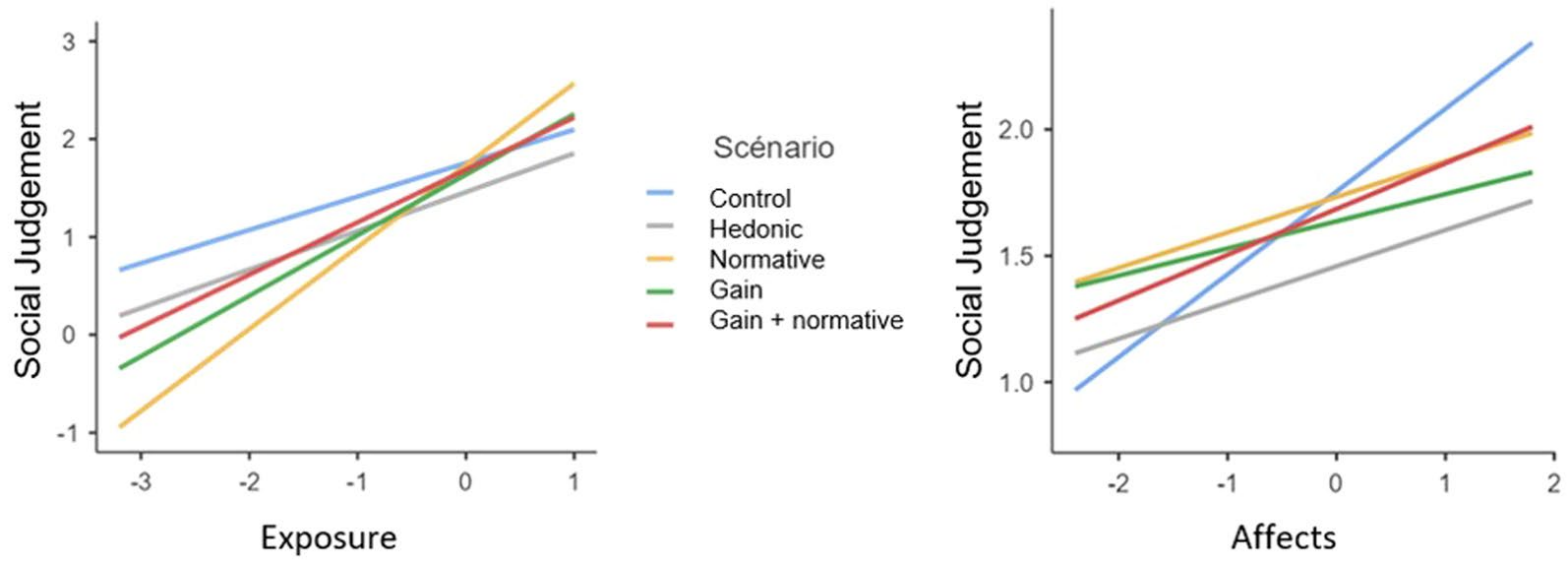
Graphs of interactions between exposure and affect factors with scenarios on social acceptability.

The same pattern of results was observed for the social acceptability of the tool. In fact, as we can see in Figure 4, when affect is low, the judgement of the tool is better among participants who have been exposed to the motivational scenarios than among those exposed to the control scenario. On the other hand, the control scenario induces a better judgement than the other scenarios when affect increased.

- Gain and Control [*t*(1,668) = −2.41, *p* = 0.001, *β* = −0.189].
- Hedonic and Control [*t*(1,668) = −2.03, *p* = 0.043, *β* = −0.158].
- Normative and Control [*t*(1,668) = −2.07, *p* = 0.039, *β* = −0.161].

In addition, we observe an interaction effect between the social acceptability measure and the exposure and affect factors (see Figure 4). At higher exposures, the Normative scenario leads to better social judgment than the:

- Control [*t*(1,668) = −3.22, *p* = 0.001, *β* = −0.25].
- Gain + normative [*t*(1,668) = −2.00, *p* = 0.045, *β* = −0.015].
- Hedonic [*t*(1,668) = −2.92, *p* = 0.004, *β* = −0.022].

However, this reverses with decreasing exposure.

Our fourth hypothesis is also partially validated. The interactions obtained indicate that risk perception and motivation improve the acceptability of the digital tool. However, the control scenario May in some cases be more useful than the motivations.

## 8 Discussion

The objective of this study was to identify the factors that encourage the use of a digital flood prevention tool and the operational levers that can be activated in an effective manner.

Firstly, with regard to experience of flooding, it can be observed that the negative consequences of flooding have a greater impact on the assessment of the digital prevention tool than the actual experience of flooding. This supports our first hypothesis. Participants who have not experienced any negative consequences as a result of flooding tend to rate the application less highly than others. This is particularly evident in direct judgments, which can be attributed to a diminished perception of the necessity for the tool in the absence of any tangible damage. In terms of indirect judgement, that is to say when respondents were asked to assess the “percentage of neighbours interested in the tool”, the estimated rate was higher for participants whose neighbourhood had been flooded than for those who had been flooded without any negative consequences. These findings align with those reported by Burningham et al. (2008), who observed that individuals who had experienced flooding with few negative consequences exhibited a diminished perception of the associated risk.

Furthermore, participants who had a relative who had experienced flooding rated the tool more favourably than those whose relatives had not experienced flooding. This result can be explained in terms of a reduction in comparative optimism, which is sensitive to experience (Helweg-Larsen and Shepperd, 2001; Weinstein, 1980). Indirect experience of a flood (i.e., through relatives), which makes it easier for participants to put themselves in the victims’ shoes, could therefore also reduce comparative optimism.

In accordance with the literature, which indicates that a best perception of risk would result in a greater motivation to protect oneself (Grothmann and Reusswig, 2006), we hypothesised that there will be a main effect of risk perception on the acceptability of the digital tool. In accordance with this one, it can be observed that an elevated perception of risk is associated with an increased acceptability of the tool. In more specific terms, it can be observed that the “affect” factor exerts the greatest positive influence on each of our acceptability measures. This is consistent with the findings of the literature, which indicate that the “Fear aroused” factor is the most significant (Slovic et al., 1980), in conjunction with the “exposure” and “knowledge” factors. This concept, refering to the notion of “Risk as feeling”, proposes that risk is perceived based on emotions and intuition, rather than through a more analytical mode of judging risk, namely “Risk as analysis”, which is based on a logical analysis of risk (Chauvin, 2014; Finucane and Holup, 2006; Slovic et al., 2004). Exposure is also a significant predictor of two measures of acceptability: the direct and the social ones. This May be associated with the fact that individuals who are highly exposed to the risk of flooding feel more vulnerable, particularly because they May live in flood-prone areas. Consequently, individuals would be more amenable to the application in order to protect themselves. Our results therefore support our second hypothesis. Moreover, this research has demonstrated the relevance of goal framing theory to the theoretical field of risk, with a particular focus on digital flood risk prevention.

The hypothesis proposed that there is a link between the acceptability of the digital tool and the salience of the goals frame in the proposed scenarios. It was hypothesised that acceptability would be higher in the presence of the activation of a goal frame. The results demonstrated equivalence between all the proposed scenarios with regard to both direct and indirect judgments of the tool. It is important to note that these measures relate to an evaluation of the tool itself and the interest it arouses among participants. Conversely, the social judgement, which pertains to the evaluation of others, exerts a differentiating effect on the scenarios. The hedonic goal scenario yielded a lower score than the other four scenarios. In other words, individuals exposed to the hedonic scenario were found to judge people using the tool as “less responsible” than those exposed to the other scenarios. It seems plausible to suggest that models of the two-dimensionality of judgement May be applicable here (Dubois and Beauvois, 2001; Fiske et al., 2002). Insofar as this tool is designed for the purpose of prevention, it can be expected that individuals will view it as a useful rather than a desirable instrument. The comments obtained during the pre-tests are consistent with this interpretation. Some subjects wrote that floods are a serious matter and that they should not be taken lightly. Consequently, the social judgement measure enables the avoidance of overly prescriptive responses to a “benevolent” tool (Lefeuvre et al., 2008) and the refinement of the judgement relating to this tool. Our analysis found only partial support for our third hypothesis.

Finally, consistent with the above, we hypothesised that the greater the exposure of participants with a higher perception of risk to motivational scenarios, the greater the acceptability of the digital tool. This fourth hypothesis was partially verified, with the results being consistent with those of the previous study. The three goals frame activated within the four scenarios (hedonic, gain and normative in isolation or combined versus control scenario) resulted in a high level of acceptability of the digital tool, regardless of affect, with a particularly notable outcome in the case of low affect. Indeed, whether in terms of direct or social judgement, it is when affect is low that exposure to the motivational scenarios leads to a more favourable judgement of the tool compared with the control scenario. Conversely, the control scenario was found to elicit a more favourable judgement than the other scenarios when affect levels increased. In essence, high affect in the context of flood risk would be sufficient to induce interest in the use of a tool to protect against the risk. Conversely, in the absence of an inherent motivation to prevent risk (low affect), exposure to a framework goal would enhance the effectiveness of using the tool.

It is similarly important to consider the risk exposure dimension, as it interacts with the goals on the social acceptability measure. It can be observed that the normative goal is less effective in the case of low exposure, whereas it is more effective than the other goals in the case of higher exposure. Given the already established and projected increase in the effects of climate change, individuals will be increasingly exposed to environmental risks, particularly flooding. Consequently, the utilisation of the normative goal frame is demonstrated to be pertinent for the instigation of a self-protection approach. This is evidenced by the *a priori* judgement of a dedicated digital tool. Results provide support for the last hypothesis (H5), which postulates that normative motivation would lead to greater acceptability of the tool. Although normative motivation does not generally improve the acceptability of the tool (H3), it becomes more effective when the perception of risk (and in particular the exposure factor) is taken into account in the analysis (H4).

The results collectively demonstrate the operational relevance of the social judgement measure in delineating the circumstances under which the tool is deemed acceptable. In particular, this “roundabout” measure enables the amplification of results in comparison to a more direct measure, and to reveal more precisely the levers of effectiveness for action with greater precision. Consequently, this type of measure can provide additional explanatory elements that would not be detected by more direct measures. Also, an understanding of how individuals utilising the application are evaluated based on their emotional state and perceived exposure to risk provides insight into how the perception of the situation emphasises the importance of the framing goals in that situation. This demonstrates that the hedonic goal, which is typically robust (Lindenberg, 2023), becomes less salient in a situation perceived as high-risk. In contrast, the normative goal is particularly salient in this situation.

There are, however, some limitations to our study. The first one is that we elected to retain Cronbach’s alpha for the knowledge factor, despite its inherent limitations. This decision was made with the objective of maintaining theoretical alignment with the psychometric paradigm. The omission of this factor would have constituted a suboptimal theoretical choice. Nevertheless, it would have been preferable for alpha to demonstrate greater reliability, thereby enhancing the precision of our results. In the future, it would be interesting to use a scale that has already been translated or to translate it in order to validate a more stable factor structure. Another weakness of this research relates to the difficulty of drafting scenarios that clearly refer to the different frame goals. Thus, although pre-tested and modified twice the material, the control scenario seems to bring out the three types of motivation moderately. We did not succeed in producing a scenario without motivations, insofar as each piece of information can lead to the activation of one of them. Similarly, it proved difficult to operationalise the hedonic scenario. We chose to present this motivation in the form of a game and demonstrated to induce hedonic motivation in individuals. To confirm this result, it would be beneficial to re-use the hedonic motivation from another angle like the fundamental needs. Insofar as this is the first time that framing goal theory has been used in a major hazard prevention context, it seems important to consider how this type of application might be refined in the future.

Future studies will be able to test the effect of comparative optimism, which we did not analyse in this study, in order to determine whether this variable could influence the acceptability of a digital prevention tool. In addition, new studies using framework goal theory in this field of research seem relevant in order to give greater validity to this theory of risk.

## 9 Conclusion

In conclusion, the tested preventive tool has been generally well received by the public. This does not guarantee that it will be used, but it represents an encouraging initial step in its development.

Upon consideration of all the data, it can be concluded that risk perception is a more accurate predictor of the acceptability of the prevention tool presented than the manipulation of the salience of frame goals. Nevertheless, the activation of certain types of motivation appears to reinforce the acceptability of the tool, particularly when the perception of risk is high. Consequently, gain and normative goals must be considered in communications relating to risk prevention by digital means. This opens up a field of reflection that could ultimately lead to a better risk culture. In order to achieve this, it is necessary to consider new ways of operationalising the frame goals in order to gain a better understanding of their possible impact in situations perceived as highly risky.

## Data Availability

The raw data supporting the conclusions of this article will be made available by the authors, without undue reservation.

## Footnotes

^1^This digital application is currently being developed as part of a project funded by *La* *Fondation MAIF* *pour la Recherche* (https://www.fondation-maif.fr/) and by *Le Syndicat Mixte du Bassin des Sorgues* (https://www.lasorgue.fr).

^2^By the society Dynata which recruited the participants: https://www.dynata.com/?lang=fr

^3^ https://www.georisques.gouv.fr/minformer-sur-la-prevention-des-risques/les-risques-naturels-en-france-chiffres-cles

^4^Consult 13/08/2024.
