# Supplementary material for: The effect of goal frame and risk perception on digital flood prevention tool acceptability
Source: Front Psychol. 2024 Sep 18;15:1454078. doi: 10.3389/fpsyg.2024.1454078 (PMC11524046; doi:10.3389/fpsyg.2024.1454078)
Supplement: Supplementary file 1 [file Table_1.DOCX]

Supplementary Material

Table 1: Regression table of the interaction between affect factor and the scenarios on direct acceptability – associated to figure 4

|  |  |  | 95% confidence interval | |  |  |  |  |
| --- | --- | --- | --- | --- | --- | --- | --- | --- |
| Names | Estimate | SE | Lower | Upper | β | df | t | p |
| Intercept | 4.747 | 0.074 | 4.602 | 4.892 | / | 1668 | 64.149 | <.001 |
| Exposure | 0.391 | 0.144 | 0.109 | 0.673 | 0.148 | 1668 | 2.718 | 0.007 |
| Affects | 0.789 | 0.084 | 0.625 | 0.954 | 0.514 | 1668 | 9.392 | <.001 |
| Knowledge | 0.018 | 0.123 | -0.223 | 0.261 | 0.008 | 1668 | 0.150 | 0.880 |
| Gain - Control | -0.065 | 0.105 | -0.270 | 0.140 | -0.042 | 1668 | -0.619 | 0.536 |
| Gain + normative - Control | 0.066 | 0.104 | -0.139 | 0.270 | 0.043 | 1668 | 0.629 | 0.530 |
| Hedonic - Control | 0.088 | 0.105 | -0.117 | 0.293 | 0.058 | 1668 | 0.844 | 0.399 |
| Normative - Control | 0.111 | 0.105 | -0.095 | 0.316 | 0.072 | 1668 | 1.056 | 0.291 |
| Affects * Gain - Control | -0.260 | 0.115 | -0.485 | -0.035 | -0.169 | 1668 | -2.269 | 0.023 |
| Affects * Gain + normative - Control | -0.297 | 0.115 | -0.522 | -0.072 | -0.194 | 1668 | -2.592 | 0.010 |
| Affects * Hedonic - Control | -0.434 | 0.114 | -0.658 | -0.210 | -0.283 | 1668 | -3.803 | <.001 |
| Affects * Normative - Control | -0.196 | 0.114 | -0.419 | 0.028 | -0.127 | 1668 | -1.718 | 0.086 |

Table 2: Regression table of the interaction between exposure and affect factor and the scenarios on social acceptability - associated to figure 5

|  |  |  | 95% confidence interval | |  |  |  |  |
| --- | --- | --- | --- | --- | --- | --- | --- | --- |
| Names | Estimate | SE | Lower | Upper | β | df | t | p |
| Intercept | 1.654 | 0.026 | 1.602 | 1.705 | / | 1668 | 62.828 | <.001 |
| Exposure | 0.546 | 0.050 | 0.447 | 0.644 | 0.273 | 1668 | 10.886 | <.001 |
| Affects | 0.180 | 0.028 | 0.125 | 0.235 | 0.155 | 1668 | 6.416 | <.001 |
| Knowledge | -0.047 | 0.043 | -0.132 | 0.037 | -0.027 | 1668 | -1.104 | 0.270 |
| Gain - Control | -0.118 | 0.083 | -0.281 | 0.0459 | -0.102 | 1668 | -1.411 | 0.158 |
| Gain + normative - Control | -0.069 | 0.083 | -0.232 | 0.094 | -0.060 | 1668 | -0.832 | 0.405 |
| Hedonic - Control | -0.296 | 0.083 | -0.459 | -0.133 | -0.256 | 1668 | -3.554 | <.001 |
| Normative - Control | -0.021 | 0.083 | -0.185 | 0.142 | -0.018 | 1668 | -0.256 | 0.798 |
| Exposure * Gain - Control | 0.278 | 0.167 | -0.050 | 0.605 | 0.139 | 1668 | 1.664 | 0.096 |
| Exposure * Gain + normative - Control | 0.194 | 0.159 | -0.117 | 0.505 | 0.097 | 1668 | 1.223 | 0.221 |
| Exposure * Hedonic - Control | 0.054 | 0.159 | -0.258 | 0.366 | 0.027 | 1668 | 0.340 | 0.734 |
| Exposure * Normative - Control | 0.496 | 0.154 | 0.194 | 0.798 | 0.248 | 1668 | 3.217 | 0.001 |
| Affects * Gain - Control | -0.220 | 0.091 | -0.399 | -0.041 | -0.189 | 1668 | -2.409 | 0.016 |
| Affects * Gain + normative - Control | -0.147 | 0.091 | -0.326 | 0.0323 | -0.126 | 1668 | -1.607 | 0.108 |
| Affects * Hedonic - Control | -0.184 | 0.091 | -0.362 | -0.006 | -0.158 | 1668 | -2.026 | 0.043 |
| Affects * Normative - Control | -0.187 | 0.091 | -0.365 | -0.009 | -0.161 | 1668 | -2.065 | 0.039 |
